# A low‐fat synbiotic cream cheese containing herbal gums, *Bifidobacterium adolescentis* and *Lactobacillus rhamnosus*: Physicochemical, rheological, sensory, and microstructural characterization during storage

**DOI:** 10.1002/fsn3.3731

**Published:** 2023-10-18

**Authors:** Reza Shahraki, Amir Hossein Elhamirad, Javad Hesari, Mostafa Shahidi Noghabi, Ahmad Pedram Nia

**Affiliations:** ^1^ Department of Food Science and Technology, Sabzevar Branch Islamic Azad University Sabzevar Iran; ^2^ Department of Food Science and Technology University of Tabriz Tabriz Iran; ^3^ Department of Food Chemistry Research Institute of Food Science and Technology (RIFST) Mashhad Iran

**Keywords:** *Lepidium perfoliatum*, low‐fat cheese, probiotic bacteria, Synbiotic

## Abstract

This study aimed to use natural herbal gums, as fat replacers, for preparing a low‐fat synbiotic cream cheese; for this purpose, the effects of *Lepidium perfoliatum* seed gum (LPSG) (1% w/w) and flaxseed gum (FG) (1% w/w) on physicochemical, rheological, organoleptic, and microstructural properties of low‐fat cream cheese containing *B. adolescentis* and *L. rhamnosus* were analyzed over a 45‐day storage period. The results indicated that adding LPSG and FG had no significant effects on acidity and pH (*p* > .05). The results also showed that full‐fat (FF) cheese samples had the highest textural (hardness (1.099–0.88), cohesiveness (0.72–0.67), springiness (1.95–1.64), adhesiveness (1.01–0.69), and spreadability (1.53–1.17)), viscosity and sensory scores (color (4.22–4.18), odor (4.13–4.09), taste (4.19–3.89), texture (4.08–3.81), and overall acceptability (4.01–3.72)) during 45‐day storage. Based on the probiotic count test, only the treated samples with LPSG + FG had a probiotic count in the standard range (6.23 cfu/g) at the end of the storage time. The outcomes of the present study indicated that the incorporation of LPSG and FG into the formulation of low‐fat synbiotic cream cheese could be an effective strategy to overcome the problems associated with fat reduction.

## Graphical abstract

The effects of *Alyssum homolocarpum* seed gum (AHSG) (1% w/w) and flaxseed gum (FG) (1% w/w) on physicochemical, rheological, organoleptic, and microstructural properties of low‐fat cream cheese containing *B. adolescentis* and *L. rhamnosus* were analyzed over a 45‐day storage period.The outcomes of the present study indicated that the incorporation of AHSG and FG into the formulation of low‐fat synbiotic cream cheese could be an effective strategy to overcome the problems associated with fat reduction.

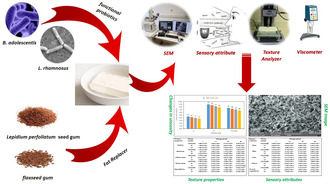

## 1 INTRODUCTION

Dairy products, which are the most basic and common foods in the human food chain, are processed from the milk of mammals as the main material and contain milk itself, ice cream, butter, cheese, yogurt, and so on. These products belong to nutrient‐dense foods, providing essential micronutrients, high‐quality protein, and energy. Moreover, the presence of bioactive compounds such as conjugated linoleic acid, oligosaccharides, minerals, vitamins, oligosaccharides, antioxidants, and peptides endow many health benefits for the consumption of dairy foods (Górska‐Warsewicz et al., 2019; Yousefi & Jafari, 2019). Cream Cheese is listed among the most important dairy products which contain a certain amount of fat from dairy or nondairy sources (Wen et al., 2021). Fat in cheese plays a key role in the cheese sensory and textural properties (Wen et al., 2021). Therefore, it is a crucial compound in cream cheese, affecting the structure, sensory attributes, texture, appearance, and stability.

Nowadays, some researchers suggest that inordinate consumption of fat contributes to the development of some diseases such as diabetes, coronary heart disease, certain types of cancer, blood pressure, weight management, and obesity (Kai et al., 2014; Kim et al., 2019). Consumers are, therefore, increasingly interested in the consumption of low‐fat dairy products. Nevertheless, the presence of fat in dairy products particularly cream cheese is very important as described earlier, and the removal of fat will induce the quality deterioration. Hence, fat replacers like herbal gums need to be utilized to mimic some physiochemical attributes and organoleptic qualities of fat.

Gums or hydrocolloids are widely utilized in food systems for different aims, including stabilizers, thickeners, and gelling agents. These can be obtained from different animal and plant resources, but gums from plant sources are more popular with consumers. Plant seeds are ancient and traditional sources for the preparation of gums (Koocheki et al., 2009). *Lepidium perfoliatum* is a plant of the *Cruciferae* family which native to Middle Eastern countries including Saudi Arabia, Pakistan, Egypt, Iraq, and Iran. Traditionally, the plant seeds are utilized for treatment of kidney stone and scurvy. Moreover, a mixture containing pharmaceuticals with *L. perfoliatum* seeds was mentioned to strengthen the nervous system and for treatment of general weakness (Eisenman et al., 2012). Several bioactive glucosinolates have been isolated from *Lepidium* species with potential antiprotozoal, anti‐inflammatory, hepatoprotective, chemoprotective, and antitumoral activities (Conde‐Rioll et al., 2018). Gum extracted from *Lepidium perfoliatum* (LPG) contains 0.18% ash, 6% moisture, 4.6% protein, and 88.23% carbohydrates (Koocheki et al., 2013) and is known as stabilizer and emollient in the food industry (Hesarinejad et al., 2014).

Linseed or flax (*Linum usitatissimum L*.) is an annual plant from *Linaceae* family. Flaxseed cake contains 3.5%–9.4% gum. Flaxseed is a heterogeneous polysaccharide, and the difference in its monosaccharide composition depends on the plant variety (Akl et al., 2020). This gum can be easily extracted and utilized as a potential food hydrocolloid, considering its thickening and emulsifying properties. Liu et al. (2018) utilized flaxseed gum as a substitute for egg white in bakery products because of its emulsifying properties. They indicated that this gum could stabilize food emulsions (at a concentration of 0.1%–5.5%.).

Probiotics, as one of the most important functional products, have beneficial effects on consumer health by influencing the body's microflora. Probiotics are live microorganisms that live synergistically in gastrointestinal (GI) tract and protect consumers against certain diseases (Tomasik & Tomasik, 2020). Prebiotics are indigestible oligosaccharides which not only improve the sensory and textural properties of food products but also help to growth and activity of probiotics. They seem to be more important than probiotics, as every individual has his/her own specific probiotics varying depending on the world regions. Thus, it is better to develop prebiotics for the digestive system of that individual rather than introducing new probiotic strains into his/her body, whether pure or with food.

However, no studies have investigated the simultaneous effect of adding herbal gums (as fat replacers) and prebiotics on the physicochemical, rheology, and sensory properties of cream cheese. Hence, the effects of LPSG and FG on low‐fat cream cheese containing *Bifidobacterium adolescentis* and *Lactobacillus rhamnosus* were studied based on evaluation of dry matter, pH, texture analysis, viscosity, viability of probiotic bacteria, microstructure of product, and sensory evaluation during 45 days of storage.

## 2 MATERIALS AND METHODS

### 2.1 Extraction of *Lepidium perfoliatum* gum (LPSG)

Firstly, the seeds of *Lepidium perfoliatum* were cleaned and soaked in distilled water at 36°C for 1 h (pH = 4; water: seed ratio of 40:1) to extract the gum. Afterward, the gum was precipitated with ethanol 96% (ethanol: gum ratio of 3:1) and dissolved in distilled water again. The prepared solution was kept in a refrigerator for 24 h and subsequently dried in a vacuum oven (Shimaz, Iran) at 40°C. Finally, the gum was ground (Phillips), screened, packaged, and stored in a cool dry condition (Koocheki et al., 2010).

### 2.2 Extraction of flaxseed gum (FG)

Firstly, the flaxseeds were cleaned and mixed with distilled water at 90°C for 3 h by using a magnetic stirrer (IKA, Germany) (pH = 5–6.7; water: seed ratio of 14:1). The obtained mixture was filtered and precipitated with ethanol 96% (ethanol: gum ratio of 3:1). The sediment was centrifuged (Hermle, Germany) at 7455 *g* for 15 min. Then, it was dried in a vacuum oven. Finally, the gum was ground (Phillips), screened, packaged, and stored in a cool dry condition (Akl et al., 2020).

### 2.3 Cheese production

At first, skimmed milk and cream were mixed followed by being pasteurized and homogenized. After that, the mixture temperature was set at 23°C in the coagulation vat, and the starter and rennet were added. The probiotic microorganisms (*B. adolescentis and L. rhamnosus*) were added to the mixture at 10^8^ cfu/g before the addition of rennet. After 12–15 h, the milk clot was formed and the pH reached approximately 4.8. The mixture was stirred and transferred to a heat exchanger (80°C) by using a pump. It was then dehydrated in the separator. Finally, less than 1% salt as well as LPSG (1%) + FG (1%) were added. The obtained cheese samples were packaged at 75°C, cooled to below 10°C, and stored at 5 ± 1°C (Miri & Habibi Najafi, 2011).

### 2.4 Physicochemical analysis

Dry matter (by the oven drying method at 105 ± 2°C) contents of cheese samples were analyzed according to the reported method by Marshall (1992). Titratable acidity (g lactic acid/100 g of sample) was assessed by titrimetric methods (Marshall, 1992). The pH meter (model Kent Hanna, Herisau, Switzerland) was utilized for the evaluation of pH value, by mixing distilled water and cheese (10:1). All results were conducted in triplicate.

### 2.5 Texture analysis

A texture analyzer (TA‐XT plus, Stable Micro Systems, Godalming, UK) equipped with a cylindrical probe (30 mm in diameter) was used to analyze the texture of the samples (hardness, cohesiveness, springiness, spreadability, and adhesiveness) which were cut into 15 × 15 × 15 mm cubes and double‐compressed after equilibration at ambient temperature. The probe penetration speed was 2 mm/s (Ningtyas et al., 2018).

### 2.6 Viscosity

According to Sołowiej et al. (2015), the apparent viscosity of low‐fat cream cheese was evaluated using a Brookfield DV II+ rotational rheometer (Brookfield Engineering Laboratories) equipped with T‐bar Spindle F and Helipath Stand. Measurements were carried out with spindle velocity of 0.5 rpm at 21°C.

### 2.7 Viability of probiotics

The viability of probiotics (*B. adolescentis and L. rhamnosus*) was determined by using MRS bile agar according to the method described by Mortazavian et al. (2006).

### 2.8 Microstructure

Cheese sample microstructures were characterized using field‐emission scanning electron microscope (FESEM, MIRA3 TESCAN, Brno, Czech Republic). At first, the samples were cut into 2 × 2 × 1 mm cubes and kept in glutaraldehyde 2.5% (w/w) for 3 h to prevent weight loss. Subsequently, the samples were immersed in liquid nitrogen for 5–10 s (Macdougall et al., 2019). The samples were chopped into 1‐mm fragments by using a cold surgical blade, which were coated with gold–palladium alloy with a thickness of 6 nm for 120 s and examined using SEM.

### 2.9 Sensory evaluation

Organoleptic assessment of the cheese samples was carried out by an experienced 20‐member panel (11 males, 9 females; age 20–25 years) (Akalin & Karaman, 2020). Thirty‐gram samples at ambient temperature (20 ± 2°C) were presented unlabeled and randomly to panelists along with palate cleansers to assess their color, odor, taste, texture, and overall acceptance in a 5‐point hedonic scale (‘1’ corresponded to ‘unacceptable’, and ‘5’ corresponded to ‘excellent ‘) (Akl et al., 2020).

### 2.10 Statistical analysis

Statistical analysis utilized in present work was conducted by a factorial experiment based on a central composite design with the factors of LPSG + FG with three replications. The data analysis was conducted by using Duncan's multiple‐range test at the probability level of 5% and by using SAS software version 1.9. Excel software was utilized to draw the graph.

## 3 RESULTS AND DISCUSSION

### 3.1 Physicochemical analysis

**FIGURE 1:**
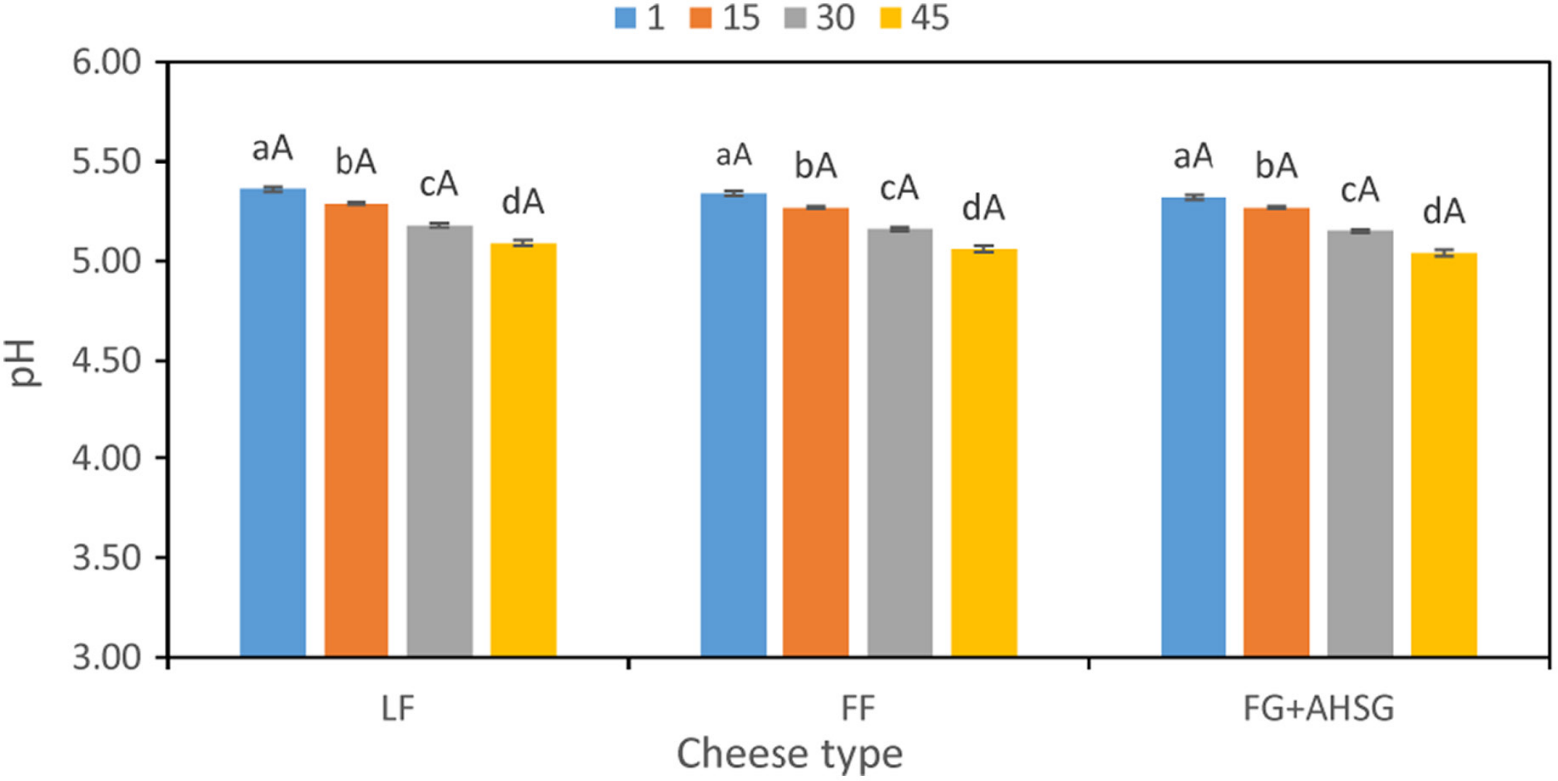
Changes in pH values of cheese samples during storage period. FF, full‐fat control sample; FG + LPSG, low‐fat sample containing flaxseed and *Lepidium perfoliatum* seed gums; LF, low‐fat control sample. ^A–C^Mean values among treatments not followed by a common letter differ significantly (*p* < .05). ^a–d^Mean values not followed by a common letter differ significantly during storage (*p* < .05).

The effects of adding LPSG + FG on pH, acidity, and dry matter of low‐fat synbiotic cream cheese are presented in Table 1 and Figure 1. According to the obtained results, adding LPSG + FG had no significant (*p* > .05) effects on pH and acidity. The results also indicated that the pH values of all cream cheese samples significantly (*p* < .05) decreased throughout keeping period. On day 1, pH values ranged between 5.36 (LF) and 5.32 (cheese containing LPSG + FG), which reached 5.09 (LF) and 5.04 (cheese containing LPSG + FG). Unlike pH values, acidity in all cheese samples significantly increased over time (Table 1). On the first day of storage, acidity ranged between 0.23 (cheese containing LPSG + FG) and 0.24 (LF, FF), which reached 0.37 (cheese containing LPSG + FG) and 0.35 (LF). Similar results have been reported by Lafta et al. (2019) on treated low‐fat cheese with Arabic gum and Ningtyas et al. (2018) on treated cream cheese with β‐glucan and phytosterol.

**TABLE 1:** Physicochemical properties of low‐fat cream cheese during keeping period.

Physicochemical properties	Cheese sample	Storage period			
		1	15	30	45
Acidity	FG + LPSG	0.23 ± 0.03^dA^	0.27 ± 0.02^cA^	0.33 ± 0.05^bA^	0.37 ± 0.06^aA^
	FF	0.24 ± 0.02^dA^	0.28 ± 0.02^cA^	0.31 ± 0.04^bA^	0.36 ± 0.05^aA^
	LF	0.24 ± 0.03^dA^	0.27 ± 0.03^cA^	0.32 ± 0.05^bA^	0.35 ± 0.05^aA^
Dry matter	FG + LPSG	72 ± 0.67^aA^	72 ± 0.51^aA^	71 ± 0.66^aA^	71 ± 0.67^aA^
	FF	60 ± 0.58^aC^	60 ± 0.67^aC^	60 ± 0.69^aC^	60 ± 0.78^aC^
	LF	66 ± 0.55^aB^	66 ± 0.77^aB^	66 ± 0.78^aB^	65 ± 0.69^aB^

^a–d^Significant changes of the samples during storage (*p* < .05). ^A–C^Significant changes compared to the treated sausages and control (*p* < .05).

Abbreviations: FF, full‐fat control sample; FG + LPSG, low‐fat sample containing flaxseed and *Lepidium perfoliatum* seed gums; LF, low‐fat control sample.

According to Table 1, the addition of FG and LPSG significantly affected the moisture content (*p* < .05). The moisture content of treated samples with LPSG + FG was significantly higher than low‐fat (LF) and full‐fat (FF) cheese samples. Increasing the moisture content of low‐fat cream cheese to equivalent to or higher than that of the full‐fat one is an important strategy for improving its textural properties. The rise in water‐binding capacity as a result of hydrocolloid addition might be the main reason for higher moisture content (Ningtyas et al., 2018). LPSG + FG acted as a water absorbent and inhibited its expulsion by developing a gel network (Gustaw et al., 2011). Ningtyas et al. (2018) also evaluated the effects of adding β‐glucan on moisture content of cream cheese and reported similar results. Zhang et al. (2021) also indicated that the combination of inulin and low‐fat probiotic cheddar cheese resulted in higher moisture content compared with control.

### 3.2 Texture analysis

#### 3.2.1 Hardness

The effects of adding LPSG + FG on hardness are presented in Table 2. According to the obtained results, the addition of LPSG + FG had a significant impact on the hardness of the samples (*p* < .05). On day 1, the hardness of treated samples with LPSG + FG was 0.89, which was significantly lower than FF (1.09) samples and higher than LF (0.28) samples (Table 2). The hardness of all samples significantly decreased throughout the storage period and reached 0.69, 0.88, and 0.16 in cream cheese containing LPSG + FG, FF, and LF, respectively. Hydrocolloids absorb the cheese moisture and give rise to its hardness by decreasing the free water content (Akl et al., 2020). Basiri et al. (2018) also reported that the presence of flaxseed mucilage in stirred yogurt increased its hardness. Ningtyas et al. (2018) also indicated that the application of β‐glucan in cream cheese resulted in higher hardness. Zhang et al. (2021) also evaluated the effects of inulin in the formula of low‐fat probiotic cheddar and showed higher hardness in treated samples.

**TABLE 2:** Texture properties of low‐fat cream cheese during keeping period.

Texture properties	Cheese sample	Storage period			
		1	15	30	45
Hardness	FG + LPSG	0.89 ± 0.07^aB^	0.85 ± 0.06^aB^	0.75 ± 0.06^bB^	0.69 ± 0.07^cB^
	FF	1.09 ± 0.09^aA^	1.06 ± 0.06^aA^	0.95 ± 0.05^bA^	0.88 ± 0.06^cA^
	LF	0.28 ± 0.05^aC^	0.26 ± 0.07^aC^	0.20 ± 0.07^bC^	0.16 ± 0.05^cC^
Springiness	FG + LPSG	1.55 ± 0.06^aB^	1.51 ± 0.07^aB^	1.40 ± 0.01^bB^	1.29 ± 0.07^cB^
	FF	1.95 0.07^aA^	1.90 ± 0.05^aA^	1.77 ± 0.07^bA^	1.64 ± 0.08^cA^
	LF	0.55 ± 0.05^aC^	0.35 ± 0.07^aC^	0.42 ± 0.06^bC^	0.33 ± 0.06^cC^
Adhesiveness	FG + LPSG	0.79 ± 0.06^aB^	0.76 ± 0.04^aB^	0.64 ± 0.05^bB^	0.54 ± 0.07^cB^
	FF	1.01 ± 0.07^aA^	0.95 ± 0.05^aA^	0.83 ± 0.04^bA^	0.69 ± 0.05^cA^
	LF	0.62 ± 0.05^aC^	0.53 ± 0.07^aC^	0.42 0.05^bC^	0.33 ± 0.07^cC^
Cohesiveness	FG + LPSG	0.69 ± 0.05^aB^	0.67 ± 0.04^aB^	0.64 ± 0.05^aB^	0.62 ± 0.06^aB^
	FF	0.72 ± 0.07^aA^	0.70 ± 0.05^aA^	0.68 ± 0.06^aA^	0.67 ± 0.07^aA^
	LF	0.23 ± 0.08^aC^	0.20 ± 0.07^aC^	0.19 ± 0.07^aC^	0.19 ± 0.06aC
Spreadability	FG + LPSG	1.34 ± 0.08^aB^	1.22 ± 0.06^bB^	1.08 ± 0.05^cB^	0.94 ± 0.05^dB^
	FF	1.53 ± 0.06^aA^	1.42 ± 0.07^bA^	1.29 ± 0.06^cA^	1.17 ± 0.06^dA^
	LF	0.90 ± 0.07^aC^	0.81 ± 0.06^bC^	0.72 ± 0.05^cC^	0.66 ± 0.04^dC^

^a–d^Significant changes of the samples during storage (*p* < .05). ^A–C^Significant changes compared to the treated sausages and control (*p* < .05).

Abbreviations: FF, full‐fat control sample; FG + LPSG, low‐fat sample containing flaxseed and *Lepidium perfoliatum* seed gums; LF, low‐fat control sample.

#### 3.2.2 Springiness

From the sensory point of view, springiness or elasticity is referred to as the degree to which the foodstuff returns to its initial state after being partially compressed in the mouth. It is mechanically defined as the amount of the deformation of a product after removing the compressive force (Fox et al., 2004). According to the obtained results, the addition of LPSG + FG had a significant impact on the hardness of the samples (*p* < .05) (Table 2). On the first day of storage, the springiness of treated samples with LPSG + FG was 1.55, which was remarkably lower than FF (1.95) samples and higher than LF (0.55) samples. The springiness of all samples significantly decreased during the keeping period and reached 1.29, 1.64, and 0.33 in cream cheese containing LPSG + FG, FF, and LF, respectively (Table 2). The hydrocolloids seem to have raised the cheese springiness by absorbing the free water of the cheese. The results of the present study are in parallel with Akl et al. (2020) who evaluated the textural attributes of fat‐free cream cheese fortified with flaxseed mucilage as a fat‐replacing agent.

#### 3.2.3 Adhesiveness

From the sensory point of view, adhesiveness is the force required to remove the food from the palate while eating, and from the mechanical point of view, it is the energy needed to overcome the adhesive forces between the food surface and the surfaces of the materials which are in contact with the food (Fox et al., 2004). According to results obtained by the present study, the addition of LPSG + FG significantly (*p* < .05) affected the adhesiveness of cream cheese (Table 2). The presence of LPSG + FG in cream cheese resulted in higher adhesiveness (day 1: 0.79; day 45: 0.54) than LF cream cheese (day 1: 0.62; day 45: 0.33). The highest adhesiveness was recorded in FF cream cheese (day 1: 1.01; day 45: 0.69). According to Table 2, all samples witnessed a decrease in adhesiveness throughout the keeping period. The results of the present study are in parallel with Ningtyas et al. (2018) who evaluated the addition of β‐glucan on textural attributes of low‐fat cream cheese. Increase in adhesiveness may be related to the ability of hydrocolloids to form gel in the presence of water and other ingredients of the product (Lazaridou & Biliaderis, 2007).

#### 3.2.4 Cohesiveness

Cohesiveness is defined as the power of the internal bonds of a food product (Ningtyas et al., 2018). Similar to the other textural properties, cohesiveness in treated samples with LPSG + FG (day 1: 0.69; day 45: 0.62) was significantly (*p* < .05) higher than LF (day 1: 0.23; day 45: 0.19) and lower than FF (day 1: 0.72; day 45: 0.67) cream cheese samples (Table 2). LPSG + FG enhanced the power of the internal bonds by absorbing the free water present in the cheese matrix. Unlike hardness, springiness, and adhesiveness, the decrease in the cohesiveness during the storage period was not significant. Zhang et al. (2021) reported that the addition of inulin to low‐fat probiotic cheddar cheese resulted in higher cohesiveness. Similar findings have been achieved by Foguel et al. (2021) and Surber et al (2021) in cream cheese.

#### 3.2.5 Spreadability

Spreadability is perceived by both the eyes and the hand and can be defined as the pressure required to achieve a uniform distribution over a surface (Daubert et al., 1998). The effects of adding LPSG + FG on spreadability are presented in Table 2. The addition of LPSG + FG had a significant impact on the spreadability of the samples (*p* < .05). On day 1, the spreadability of treated samples with LPSG + FG was 1.34, which was significantly lower than FF (1.53) samples and higher than LF (0.90) samples (Table 2). The spreadability of all samples significantly decreased throughout the storage period and reached 0.94, 1.17, and 0.66 in cream cheese containing LPSG + FG, FF, and LF, respectively. The results of the present study are in agreement with Ningtyas et al. (2018) who evaluated the addition of β‐glucan on textural attributes of low‐fat cream cheese. The authors indicated that the low‐fat cream cheese containing β‐glucan was more spreadable than low‐fat control cheese.

### 3.3 Viscosity

**FIGURE 2:**
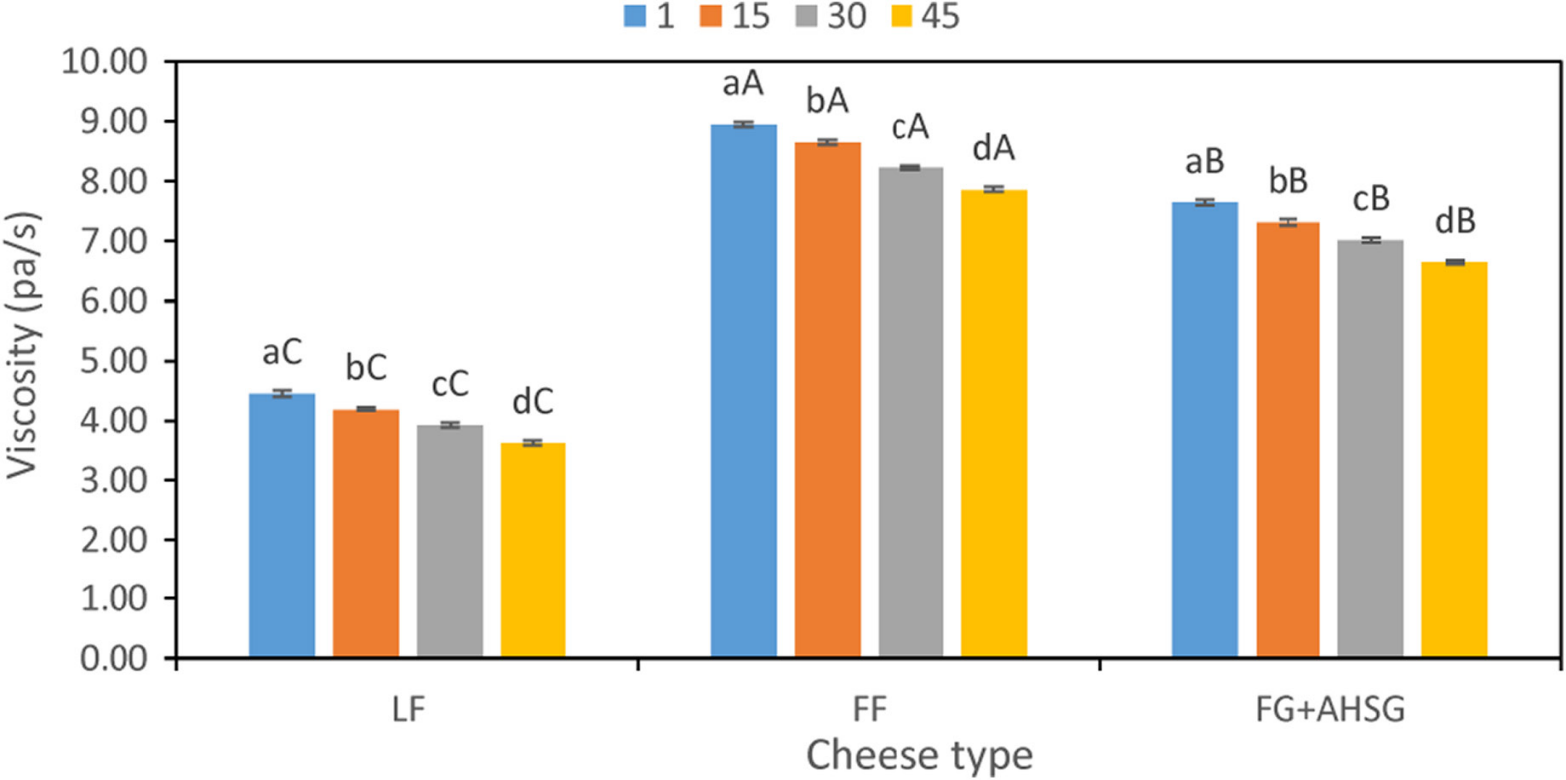
Changes in viscosity of cheese samples during storage period. FF, full‐fat control sample; FG + LPSG, low‐fat sample containing flaxseed and *Lepidium perfoliatum* seed gums; LF, low‐fat control sample. ^A–C^Mean values among treatments not followed by a common letter differ significantly (*p* < .05). ^a–d^Mean values not followed by a common letter differ significantly during storage (*p* < .05).

The outcomes of cheese apparent viscosity are illustrated in Figure 2. The results indicated that the viscosity of all cheese samples significantly decreased during storage period. The results also indicated that FF cheese samples had the highest viscosity throughout keeping period, treated samples with LPSG + FG were recorded as the second, and the LF samples were recorded as the third (Figure 2). The results of present study are in agreement with Ghasempour et al. (2012) who produced probiotic low fat yogurt containing fat replacers (Zedo gum). Similar results were also reported by Mashayekhi et al. (2016) on enriched whipped cream with sodium caseinate and Persian gum. Seyfoddin et al. (2016) showed that the addition of *Lepidium perfoliatum* seed gum to low‐fat mayonnaise potentially increased its viscosity compared with low‐fat samples which are in parallel with present study.

### 3.4 Probiotic viability

**FIGURE 3:**
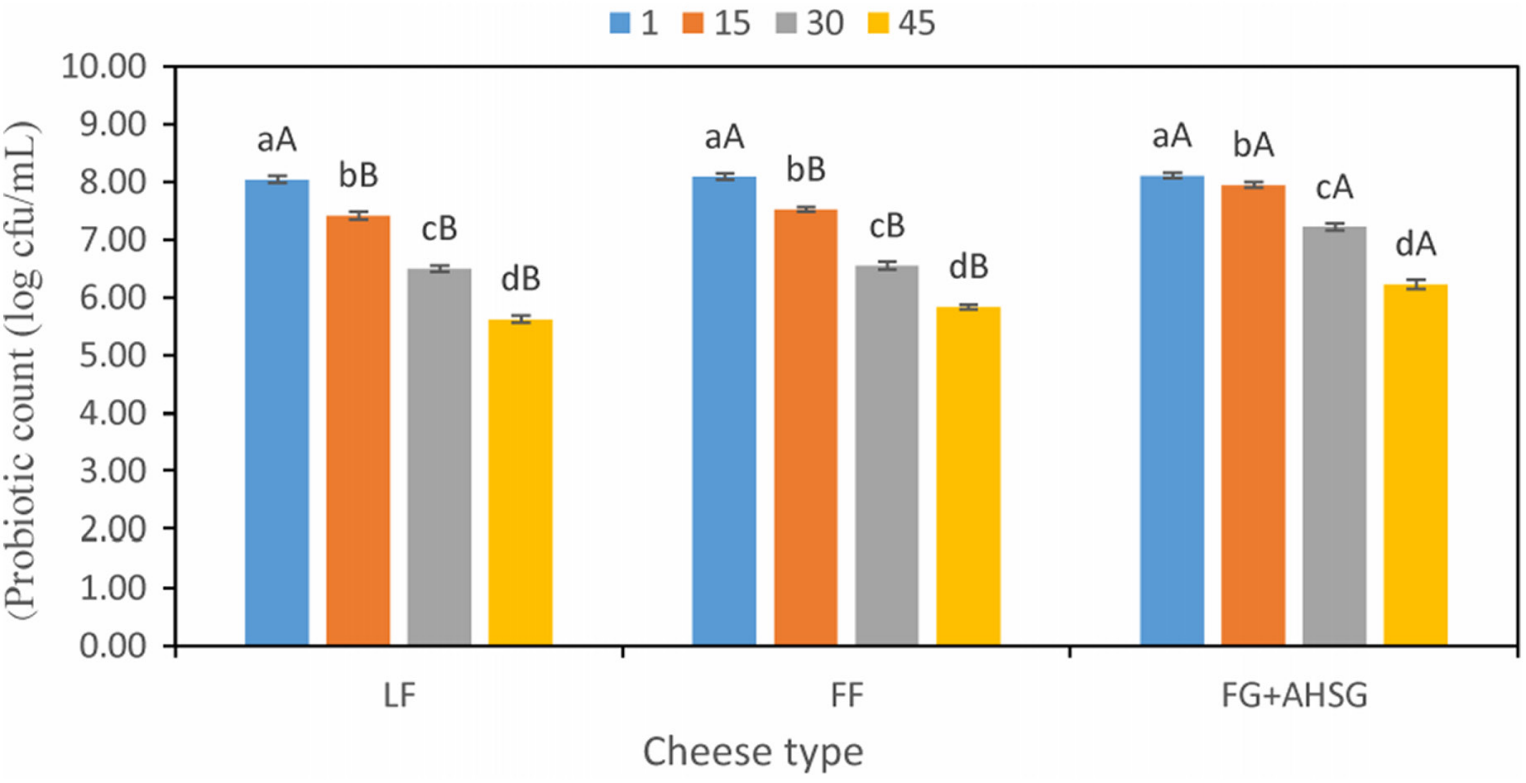
Changes in probiotics count during storage period. FF, full‐fat control sample; FG + LPSG, low‐fat sample containing flaxseed and *Lepidium perfoliatum* seed gums; LF, low‐fat control sample. ^A–C^Mean values among treatments not followed by a common letter differ significantly (*p* < .05). ^a–c^Mean values not followed by a common letter differ significantly during storage (*p* < .05).

The viability of *B. adolescentis* and *L. rhamnosus* in cream cheese samples during the 45 days of storage is indicated in Figure 3. The results showed that during the storage period, the probiotic count was significantly reduced (*p* < .05) and reached 5.63, 5.84, and 6.23 cfu/g in the LF, FF, and samples containing LPSG + FG, respectively, at the end of storage. Therefore, only the cheese samples containing LPSG + FG could be considered as probiotic cream cheese, because of having a sufficient amount of the bacteria. According to Figure 3, treated samples with LPSG + FG had the highest amount of probiotics during keeping period. The viability of probiotic bacteria depends on different factors including the treatment type, the extent of compound metabolization as well as the pH reduction owing to the consumption of prebiotics (Boylston et al., 2004). It has been found that the application of prebiotics in food products brings about the viability of probiotics to extend. As a result, it can be expressed that FG and LPSG could appropriately act as prebiotic components. Ghaderi‐Ghahfarokhi et al., 2020 indicated that the use of tragacanth in low‐fat yogurt improved the viability of *Lactobacillus casei*. Similarly, Jirsaraei et al. (2016) realized that inulin and lactulose increased the viability of *Lactobacillus casei* in probiotic UF cheese. Zhang et al. (2021) also understood that the inclusion of inulin in the formula of low‐fat probiotic cheddar cheese led to an increase in the activity and growth of *Lactobacillus plantarum*.

### 3.5 Microstructure

**FIGURE 4:**
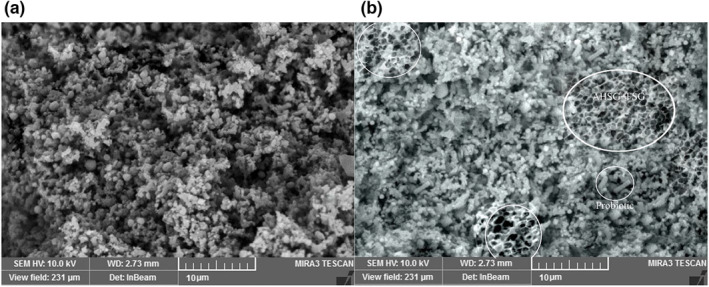
SEM images of cream cheese samples (Mag = 3.00 KX). (a): control, (b): treated samples with 1% FG + 1%LPSG.

The SEM images of the control and treated samples with LPSG + FG are presented in Figure 4. According to the obtained results the control cream cheese had spherical fat globules dispersed in the casein matrix. A dense protein network can be observed in the control cheese samples. On the other hand, the samples containing FG + LPSG had less compact structures, owing to the function of these gums as active fillers. When fat replacers are homogeneously dispersed in the casein matrix, they break down the casein bonds and develop the porous matrix of cream cheese (Gustaw et al., 2011). The results of the present study are in parallel with Rahimi et al. (2007) who evaluated the SEM images of low‐fat Iranian white cheese containing tragacanth gum as fat replacer. In Figure 4, the *L. rhamnosus* can be apparently seen as a short rod‐shaped structure. Casein aggregation was more obvious in the cream cheese samples containing the probiotic bacterium, compared with the control which might be related to the higher amount of the exopolysaccharide (EPS) produced by the starter culture and *L. rhamnosus* (Ningtyas et al., 2018). Casein, fat globules, and EPS developed a more apparent three‐dimensional network in the treated samples than in the control, resulting in the creation of a dense cluster of casein. Similar results have been obtained by Hassan et al. (2003) in low‐fat cream cheese containing β‐glucan, phytosterol, and *L. rhamnosus* (encapsulated and nonencapsulated). As can be seen in Figure 4, the gums were relatively evenly distributed in the cheese matrix. Additionally, the broader distribution of the gums in the matrix was clearly obvious when their concentrations increased.

### 3.6 Sensory properties

Organoleptic attributes, which highly affected the marketing of products, reflect the overall quality of products as well as consumer's preference to a certain extent. With regard to Table 3, all organoleptic attributes unless color and odor declined remarkably among the keeping time. On day 1, the results indicated that FG + LPSG significantly affected all sensory attributes except odor. The color results indicated that at day 45, FF cheese samples showed significantly higher scores (4.18) compared to treated samples with FG + LPSG (3.92) and LF samples (3.87). The taste, texture, and overall acceptability also showed similar trends. The outcomes of the present work revealed that until day 15, the decrease in taste, texture, and overall acceptability scores was not significant, unlike color and odor scores. As expected, decrease in fat content potentially leads to decrease in organoleptic attributes of cheese samples, which is in agreement with results obtained by Zalazar et al. (2002) on low‐fat soft cheeses and Romeih et al. (2002) on low‐fat white‐brined cheese. The results of present work are in parallel with Akl et al. (2020). The authors indicated that the use of flaxseed mucilage as a fat replacer in fat‐free cream cheese improved its taste and texture acceptability. Aydinol and Ozcan (2018) indicated that the addition of fat replacers in formulation of reduced fat Labneh cheese potentially leads to higher texture and taste scores compared with control. Similarly, Rashidi et al. (2018) indicated that the application of fat replacers (xanthan–guar mixture) could improve the taste, texture, and general acceptance of low‐fat UF feta cheese. Zhang et al. (2021) reported that the general acceptance of low‐fat cheddar cheese can be significantly increased in the presence of fat replacers.

**TABLE 3:** Sensory attributes of low‐fat cream cheese during keeping period.

Sensory attributes	Cheese sample	Storage period			
		1	15	30	45
Color	FG + LPSG	3.98 ± 0.06^aB^	3.99 ± 0.06^aB^	3.95 ± 0.05^aB^	3.92 ± 0.07^aB^
	FF	4.22 ± 0.07^aA^	4.20 ± 0.05^aA^	4.24 ± 0.04^aA^	4.18 ± 0.05^aA^
	LF	3.92 ± 0.05^aB^	3.89 ± 0.07^aB^	3.88 ± 0.06^aB^	3.87 ± 0.04^aB^
Odor	FG + LPSG	4.12 ± 0.06^aA^	4.10 ± 0.06^aA^	4.13 ± 0.07^aA^	4.08 ± 0.04^aA^
	FF	4.13 ± 0.05^aA^	4.14 ± 0.05^aA^	4.11 ± 0.04^aA^	4.09 ± 0.03^aA^
	LF	4.10 ± 0.04^aA^	4.12 ± 0.05^aA^	4.11 ± 0.05^aA^	4.13 ± 0.05^aA^
Taste	FG + LPSG	3.45 ± 0.07^aB^	3.37 ± 0.06^abB^	3.25 ± 0.06^bB^	3.12 ± 0.05^cB^
	FF	4.19 ± 0.06^aA^	4.11 ± 0.05^abA^	4.00 ± 0.05^bA^	3.89 ± 0.07^cA^
	LF	3.32 ± 0.07^aC^	3.27 ± 0.04^abC^	3.15 ± 0.06^bC^	3.01 ± 0.07^cC^
Texture	FG + LPSG	3.19 ± 0.05^aB^	3.14 ± 0.05^abB^	3.08 ± 0.0^bB^	2.90 ± 0.04^cB^
	FF	4.08 ± 0.06^aA^	4.00 ± 0.05^abA^	3.91 ± 0.05^bA^	3.81 ± 0.06^cA^
	LF	1.65 ± 0.07^aC^	1.58 ± 0.04^abC^	1.50 ± 0.06^bC^	1.35 ± 0.05^cC^
Overall‐acceptability	FG + LPSG	3.27 ± 0.07^aB^	3.19 ± 0.07^abB^	3.12 ± 0.07^bB^	3.00 ± 0.07^cB^
	FF	4.01 ± 0.06^aA^	3.95 ± 0.06^abA^	3.87 ± 0.06^bA^	3.72 ± 0.06^cA^
	LF	2.27 ± 0.04^aC^	2.20 ± 0.0^abC^	2.14 ± 0.04^bC^	2.02 ± 0.04^cC^

^a–c^Significant changes of the samples during storage (*p* < .05). ^A–C^Significant changes compared to the treated sausages and control (*p* < .05).

Abbreviations: FF, full‐fat control sample; FG + LPSG, low‐fat sample containing flaxseed and *Lepidium perfoliatum* seed gums; LF, low‐fat control sample.

## 4 CONCLUSION

With the increasing demand for low‐fat cheese, it is of importance to make equivalent structures using fat replacers. In order to promote the nutritional value of cream cheese along with retaining its qualitative and nutritional properties, the formulation of low‐fat synbiotic cream cheese was optimized using fat replacers including LPSG + FG. The results revealed that the application of the fat replacers had no significant effects on the cheese pH and acidity, while increasing its moisture content. The fat replacers positively affected (*p* < .05) the textural and organoleptic properties of the cheese compared with low‐fat cream cheese. Furthermore, at the end of storage, only the samples containing the LPSG + FG had a sufficient amount of the probiotic bacteria. According to the obtained results, synbiotic low‐fat cream cheese formulated with herbal gums and probiotic bacteria can be a suitable alternative to all kinds of high‐fat cream cheeses, because in addition to imitating the textural and sensory characteristics of cream cheese, due to the use of skimmed milk and fat removal, it has significant dietary features and can be considered as a functional product. It is suggested to carry out additional studies in order to inoculate probiotic bacteria by microencapsulation method to increase the shelf life of probiotic microbial flora and to investigate it in the simulated environment of the digestive system.

## AUTHOR CONTRIBUTIONS

**Reza Shahraki:** Writing – original draft (lead); writing – review and editing (supporting). **Amir Hossein Elhamirad:** Conceptualization (lead); writing – review and editing (lead). **javad hesari:** Formal analysis (equal). **Mostafa Shahidi Nighabi:** Writing – original draft (equal). **Ahmad Pedramnia:** Writing – review and editing (supporting).

## CONFLICT OF INTEREST STATEMENT

The authors declare no conflict of interest relevant to this article.

## ETHICS STATEMENT

This article does not cover any human or animal studies conducted by any of the authors. Not applicable.

## Data Availability

Data are available upon request from the authors.
